# Supercritical Carbon Dioxide Extracts of *Cordyceps sinensis*: Chromatography-based Metabolite Profiling and Protective Efficacy Against Hypobaric Hypoxia

**DOI:** 10.3389/fphar.2021.628924

**Published:** 2021-08-26

**Authors:** Jigni Mishra, Washim Khan, Sayeed Ahmad, Kshipra Misra

**Affiliations:** ^1^ Save The Environment, Gurugram, India; ^2^ Bioactive Natural Products Laboratory, Department of Pharmacognosy and Phytochemistry, School of Pharmaceutical Education and Research, Jamia Hamdard, New Delhi, India; ^3^ National Center for Natural Products Research, The University of Mississippi, Oxford, MS, United States

**Keywords:** *Cordyceps sinensis* (Berk) Sacc., GC-MS, HPTLC, metabolomics, supercritical fluid extract, hypobaric hypoxia (HH)

## Abstract

The toxicity and disposal concerns of organic solvents used in conventional extraction purposes has entailed the need for greener alternatives. Among such techniques, supercritical fluid extraction (SFE) has gained popularity by yielding extracts of high purity in a much faster manner. Carbon dioxide (CO_2_) is generally preferred as a supercritical solvent because of its lower temperature requirements, better diffusivity and easy removal. The present study describes the characterization of supercritical CO_2_ extracts of Indian variety of *Cordyceps sinensis* (CS)- a high-altitude medicinal mushroom widely revered in traditional medicine for its extensive anti-hypercholesterolemic, anti-inflammatory, anti-proliferative and energy-enhancing properties. Experimental parameters viz. 300 and 350 bar of extraction pressure, 60°C of temperature, 0.4°L/h CO_2_ of flow rate and use of 1% (v/v) of ethanol as entrainer were optimized to prepare three different extracts namely, CSF1, CSF2 and CSF3. High-performance thin-layer chromatography (HPTLC) was used for assessing the quality of all the extracts in terms of cordycepin, the pivot biomarker compound in CS. Characterization by HPTLC and GC-MS confirmed the presence of flavonoids and nucleobases and, volatile organic compounds (VOCs), respectively. The chromatographic data acquired from metabolite profiling were subjected to chemometric analysis in an open source R studio which illustrated interrelatedness between CSF1 and CSF2 in terms of two major principal components. i.e. Dim 1 and Dim 2 whose values were 40.33 and 30.52% in variables factor map plotted using the HPTLC-generated retardation factor values. The factor maps based on retention times of the VOCs exhibited a variance of Dim 1 = 43.95% and Dim 2 = 24.85%. Furthermore, the extracts demonstrated appreciable antibacterial activity against *Escherichia coli* and *Salmonella typhi* by generation of reactive oxygen species (ROS), protein leakage and efflux pump inhibition within bacterial pathogens. CSFs were elucidated to be significantly cytoprotective (*p* < 0.05) in a simulated hypobaric hypoxia milieu (0.5% oxygen). CSF2 showed the best results by effectively improving the viability of human embryonic kidney (HEK 293) cells to 82.36 ± 1.76% at an optimum dose of 100 µg/ml. Levels of hypoxia inducible factor-1 alpha (HIF-1α) were modulated four-fold upon supplementation with CSF2. The results collectively evinced that the CSF extracts are substantially bioactive and could be effectively utilized as mycotherapeutics for multiple bioeffects.

## Introduction

The increasing popularity of alternative healthcare has opened new dimensions for extraction of high value-added products from natural sources like medicinal plants and mushrooms. Though conventional extraction techniques like maceration, Soxhlet extraction, hydro-distillation, pressurised liquid extraction, etc. have been adopted for product development, however, certain disadvantages such as high operational energy requirements, use of expensive and toxic organic solvents, disposal concerns of the solvents, lesser selectivity in extraction and loss of volatile compounds have entailed the search for an efficient, environment friendly extraction approach (Malaman et al., 2011). One such approach is “supercritical fluid extraction” that utilizes supercritical fluids that above their critical points exhibit dual liquid-like and gas-like nature. In this manner, supercritical fluids are capable of solvent power with negligible surface tension as well as excellent mass transfer properties (Pronyk and Mazza, 2009). Although supercritical water and carbon dioxide (CO_2_) have garnered major attention amongst researchers for extracting superior quality cosmeceuticals, essences, fragrances, pharmaceuticals from medicinal herbs, CO_2_ happens to be advantageous in industrial bioprocessing owing to its comparatively moderate supercritical temperature, better diffusivity, low viscosity and easy recovery in addition to being an economical solvent, nonflammable and recyclable gas (Montesantos and Maschietti, 2020). Previous literature evidence that CO_2_ is contemplated to be a better supercritical fluid than other solvents like water (Kumoro et al., 2010). In case of water, the temperature requirement is relatively much higher than that for CO_2_. For instance, in a study describing the preparation of tumuji oil, a temperature of 500°C was used (Meng et al., 2006). Similarly, for extracting Huadian oil shale using water in supercritical state, the temperature required was 373°C (Hu et al., 1999). Such higher temperatures are not suitable for extraction of bioactive compounds from natural sources because of their thermolabile properties. In this perspective, CO_2_ is preferred as a solvent especially in the areas of functional food and pharmacological products because of its moderate supercritical points (Norodin et al., 2016; Bittencourt et al., 2021). Therefore, in the present study, supercritical CO_2_ extraction was employed to prepare bioactive extracts from Indian high-altitude variety of medicinal mushroom, *Cordyceps sinensis*, popularly known as “keera jhari” in the Kumaon region of Indian Himalayas, from where the particular variety used in current study is sourced (Pal and Misra, 2018).

*C. sinensis* has been extensively revered as “ethnomedicine” in ancient medicinal systems like Ayurveda and traditional Chinese medicine, due to its multitude of health benefits like anti-inflammatory, antioxidant, cardioprotective, endurance enhancing, hepatoprotective, etc. effects. Recent research has established evidence-based pharmacological effects by demonstrating the potential of *C. sinensis* as anti-proliferative, immunomodulatory, cardiostimulant and the like (Holliday and Cleaver, 2008). It has been widely acclaimed in previous literature for its efficacy in modulating physiological systems including the circulatory, immune, hematogenic, cardiovascular, respiratory and glandular systems of the human body under various stressful conditions (Koh et al., 2003; Yi et al., 2004; Jordan et al., 2008; Ahmed et al., 2012; Nie et al., 2013; Yan et al., 2013; Rajput et al., 2021). The substantial therapeutic value of *C. sinensis* can be attributed to its rich chemical composition encompassing glycosides, lipids, phenolics, proteins and peptides, sterols and so forth (Rajput et al., 2020).

Although the restorative potential of *C. sinensis* has been harnessed for formulation of numerous medicinal products and supplements, however its role in the management of high-altitude-induced maladies namely, acute mountain sickness (AMS), chill blains, high-altitude cerebral edema (HACE), high-altitude pulmonary edema (HAPE) and hypothermia are relatively untapped. Only few researchers have reported the considerable potential of *C. sinensis* in amelioration of adverse effects brought about by high-altitude hypoxia. For instance, aqueous alcoholic extracts of *C. sinensis* have restorative effects to treat hypoxic pulmonary hypertension by downregulating the expression of PCNA, c-fos and c-jun (Gao et al., 2010). Chen et al. (2014) have shed light on the vital role of a supplement composed of *Rhodiola crenulata* and *C. sinensis* in augmenting aerobic performance of healthy male subjects during a 2-wk high-altitude training regime where significant normalization of parasympathetic nervous system action and a prolonged exhaustive run time was observed. In studies carried out by our group, it has been observed that aqueous extracts and phenolic rich fractions of Indian Himalayan variety of *C. sinensis* have substantially averted the debilitating impact of hypobaric hypoxia *in vitro* in various mammalian cell lines namely, A549, HT22, HEK 293 cell lines and, *in vivo* in male Sprague Dawley rat models (Singh et al., 2013; Pal et al., 2015; Rajput et al., 2020). To further establish the promising adaptogenic potential of *C. sinensis* in a hypobaric hypoxia milieu, supercritical fluid extracts were prepared from *C. sinensis* (“CSF extracts”) using supercritical CO_2_ as main solvent and ethanol as entrainer. The extracts were well characterized by high-performance thin layer chromatography (HPTLC) in terms of cordycepin, flavonoids and nucleobases and in terms of volatile organic compounds (VOCs) by gas chromatography-mass spectrometry (GC-MS). Chromatography-based metabolomics using principal component analysis was carried out to group the extracts based on their metabolite content. Cognate bioactivities viz., free radical scavenging, ferric ion reduction and bactericidal action against *Escherichia coli* (*E. coli*) and *Salmonella typhi* (*S. typhi*) were revealed. These are common pathogenic bacteria whose invasion in human host cells is reportedly aggravated under hypoxia conditions (Ding et al., 2001; Jennewein et al., 2015). Since the main objective of the present study was to establish the efficacy of supercritical CO_2_ extracts of *C. sinensis* for recuperation in low oxygen tension, hence the selection of the abovementioned bacterial strains was considered relevant. Furthermore, the protective role of the fractions in resolving hypoxia-instigated concerns were thoroughly studied *in vitro* in HEK 293 cell lines where CSF extract significantly recuperated cellular viability under oxygen deficient conditions and also modulated levels of major hypoxia transcription factor, i.e., hypoxia inducible factor-1 alpha or, “HIF-1α.”

The novelty of the present manuscript resides in the fact that the protective action of supercritical CO_2_ extracts of *C. sinensis* against hypobaric hypoxia has been addressed for the very first time. Reports pertaining to hypoxia protective action are not available for supercritical extracts of any species of *Cordyceps* genus. In our earlier studies, anti-hypoxia effects of phenolic fractions and aqueous extracts (prepared by accelerated solvent extraction) of Indian Himalayan variety of *C. sinensis* were confirmed (Singh et al., 2013; Rajput et al., 2020). However, this is the very first time that supercritical carbon dioxide extracts of this variety have been researched. This is the first study of its kind that provides a detailed insight into the protective role of *C. sinensis* supercritical CO_2_ extracts against a hypobaric hypoxia milieu. These observations, along with metabolomics performed on chromatography profiling can further be taken up as a lead for value-added product development, especially against high-altitude malaises.

## Materials and Methods

### Chemicals and Reagents

Ultra-pure, molecular grade chemicals from Sigma-Aldrich (St. Louis, MO, United States) were used in the entire study. Solvents were purchased from Merck (Rahway, NJ, United States). Water used throughout was of Millipore grade (Merck, United States). Powder of *C. sinensis* was commercially procured from Aryan Mushroom (Batch No. CO-1601; Delhi, India). The average particle size of *C. sinensis* powder was 300 μm, as determined using Supra scanning electron microscope (Carl Zeiss, Germany). Bacterial strains used were *E. coli* (ATCC 9837) and *S. typhi* (clinical isolate from All India Institute of Medical Sciences, Delhi, India). HEK 293 cell line was procured from National Centre for Cell Science (Pune, India). Nutrient agar, nutrient broth and antibiotic solutions used were from HiMedia^®^ (Mumbai, India). Dulbecco’s minimal essential medium (DMEM) used in cell culture studies was procured from HiMedia^®^ (Mumbai, India).

### Preparation of *C. sinensis* Supercritical Extracts

The supercritical fluid extracts of *C. sinensis* collectively referred to as “CSF extracts” were prepared in SFE-500R system (Thar, Pittsburgh, United States), by modifying a methodology reported earlier (Miao et al., 2010). The instrument had two separate feed lines for CO_2_ and the entrainer (here, ethanol). The optimum pressure and temperature for preparing CSF extracts in the current study were finalized after evaluating the yields (%, w/w) obtained from a combination of four extraction pressures viz., 200, 250, 300 and 350 bar and three temperatures of 40, 50 and 60°C. The afore stated pressures and temperatures were selected by keeping in consideration the type of bioactive molecules (flavonoids, nucleobases and VOCs) being targeted putatively in the extracts (He et al., 2005; Liza et al., 2010; Shan et al., 2012). CO_2_ flow rate was maintained at 0.4 L/h. Ethanol 1% (v/v) was used as an entrainer/co-solvent. Amongst all the combinations of extraction pressures and temperatures tested, the ones giving extracts of highest yields (“CSF1” followed by “CSF2”) were selected as the optimum extraction conditions. Maintaining the same extraction parameters as “CSF1,” but without the use of entrainer, resulted in extraction of “CSF3.” In the experimental procedure, CO_2_ from the cylinder was released *via* a cooling bath in order to hold a constant temperature for ensuring a constant feeding rate of the pump. Subsequently, the mixture of *C. sinensis* powder, supercritical CO_2_ and ethanol for CSF1 and CSF2 and *C. sinensis* powder and only supercritical CO_2_ in case of CSF3 were contacted in a mixer. The temperature of the reaction mixture was controlled by a heating jacket. At extraction pressure of 300 bar, an extraction time of 2 h was maintained for acquiring CSF1 and CSF3. For CSF2, the extraction pressure was higher i.e., 350 bar, hence the time was reduced to 1.5 h. After an extraction time period of 2, 1.5 and 2 h for CSF1, CSF2 and CSF3 separately, the ensuing extracts were collected in the separation vessel. All the procedural parameters were regulated by the instrument console.

### Characterization of the CSF Extracts by HPTLC

Chemical characterization of the CSF extracts in terms of cordycepin, nucleobases and flavonoids was accomplished by HPTLC. The entire HPTLC analysis was accomplished on a CAMAG system (Muttenz, Switzerland) consisting of a Linomat 5 sample applicator, TLC Scanner 3 and Reprostar 3 documentation system. The samples were applied by means of a Hamilton microsyringe (100 µL) on 20 × 10 cm glass backed silica gel 60 F_254_ HPTLC plates (Merck, Billerica, MA, United States). Spectral scanning was done using winCats software (version 1.4.4.6337).

### HPTLC Analysis of Cordycepin

Quality assessment of the supercritical CO_2_ extracts was performed by characterizing them in terms of the major biomarker metabolite of *C. sinensis*, i.e. “cordycepin” or 3’-deoxyadenosine. Here, 0.5 mg of cordycepin was dissolved in 1 ml of methanol to form the cordycepin standard solution. For sample preparation, 20 mg of each CSF extract was dissolved in 10 ml of methanol thus, forming final sample concentration of 2 mg/ml. Three different application volumes (0.5, 1 and 2 µl) of cordycepin standard solution and 20 µl of each sample was applied onto separate lanes of uniform band thickness of 6 mm on a silica gel plate. The plate was developed using a mobile phase comprising chloroform, methanol and water in a ratio of 52: 7: 0.5 (v/v/v) (Sari et al., 2016). The plate was developed in a CAMAG twin-trough vertical development chamber, pre saturated with mobile phase. The solvent front was maintained till 85 mm. Thereafter, the plate was subjected to densitometric scanning at 254 nm in absorption mode, with deuterium as the light source. Slit width was 6 × 0.3 µm. Quantities of cordycepin in the three CSF extracts were computed from the corresponding peak areas generated by winCats software.

### HPTLC Analysis of Nucleobases

For quantification of nucleobases, specifically, thymine, uracil, adenine, cytosine, guanine and guanosine present in CSFs by HPTLC, stock solutions of the standard nucleobases were prepared by dissolving 0.5 mg of each standard in 1 ml of methanol and vortexing the same for 10 min. A standard mixture comprising equal volume from each of these six standard nucleobase solutions was prepared. CSF samples were prepared as mentioned in previous paragraph. Then, 6, 7 and 8 µl of the standard mixture and 20 µl of individual CSF extract were applied on silica gel plates as mentioned above. The constituent nucleobases were identified by developing the plate in a solvent system composed of dichloromethane, methanol and formic acid in a ratio of 8: 2.25: 0.8 (v/v/v) (Mishra et al., 2018b). All the analytical parameters for separation and quantification of nucleobases were retained as described in the previous section for cordycepin.

### HPTLC Analysis of Flavonoids

A standard mixture of flavonoids containing equal volume of quercetin, gallic acid, ascorbic acid and rutin was made up in a similar manner as that for nucleobases. Flavonoids present in the CSF samples were separated and quantitated using a mobile phase constituted of ethyl acetate: dichloromethane: formic acid: glacial acetic acid: methanol in a ratio of 10:10:1:1:2 (v/v/v/v/v) (Bhardwaj et al., 2015). Rest analytical and detection parameters were maintained as described in the previous sections.

### Characterization of the CSF Extracts by GC-MS

Detection of VOCs present in CSFs was carried out upon a fused silica stationary phase Rtx-5MS, having dimensions 30 m × 0.25 mm, 2 μm. GC-MS analysis was conducted using a Shimadzu QP2010 system (Kyoto, Japan), equipped with an AOC-20i autosampler. The following analytical parameters were set: sample injection volume: 1 μl; injection temperature: 260°C; purge rate: 3 ml/min; gas flow rate: 1.21 ml/min; column temperature: initial at 60°C for 2 min, then ramped at a rate of 10°C/min till 280°C; total run time: 27 min. An electron beam of 70 eV was used for ionization of samples. VOCs detected in the supercritical extracts were confirmed by matching their respective mass spectra and retention times with existing entries in NIST library (Cui et al., 2017).

### Metabolomics Studies by Principal Component Analysis

The metabolite profiling acquired from HPTLC and GC-MS analyses were utilized as input data to derive relatability patterns among the CSF extracts by means of chemometrics. Here, principal component analysis (PCA) for data reduction was realized in an open source R studio: A Language and Environment for Statistical Computing (R Foundation for Statistical Computing, Vienna, Austria (http://cran.r-project.org/). The entire datasets comprising retardation factors and peak areas of various nucleobases and flavonoids in CSF extracts in case of HPTLC, and the retention times and area percentages of constituent VOCs from GC-MS output were represented in terms of two principal components denoted as “Dim1” and “Dim2” in separate variables factor maps generated from PCA. This facilitated drawing interrelatedness patterns among the CSF extracts while ensuring data compression (Bhardwaj et al., 2017).

### Antioxidant Potential of the CSF Extracts

The antioxidant potency of *C. sinensis* supercritical CO_2_ extracts was adjudged by evaluating their free radical scavenging potential for 2,2’-azinobis-3-ethylbenzothiazoline-6-sulphonic acid (ABTS) and 2,2-diphenyl-1-picryl-hydrazyl-hydrate (DPPH) radicals as well as by determining the ferric ion reducing antioxidant power (FRAP), as detailed below.

### DPPH Assay

Various concentrations viz., 10, 5, 2.5, 1.25 and 1 mg/ml of the supercritical fluid extracts were prepared. 24 mg of DPPH powder was dissolved in 100 ml of ethanol. 10 ml of this solution was mixed well with 45 ml of methanol. Absorbance was adjusted to 1.1 ± 0.02 units at 515 nm to form the working solution. Then, 10 µl of sample was added to 190 µl of this working solution and left undisturbed for 2 h to allow proper reaction with the stable DPPH radical. A change in colour from deep violet to light yellow was measured at 515 nm. Results were represented in terms of micromole Trolox equivalent per gram of extract (µM TE/g of extract) (Mishra et al., 2018a).

### ABTS Assay

ABTS free radical scavenging assay was determined as reported elsewhere (Sharma et al., 2015). Trolox ranging from 800 to 50 µM were taken as standards to plot the standard curve. Different concentrations viz., 10, 5, 2.5, 1.25 and 1 mg/ml of all the CSF extracts were prepared. ABTS free radical scavenging values of CSF extracts were expressed as μM TE/g of extract. Absorbances were recorded at 734 nm.

### FRAP Assay

FRAP values of CSF extracts were determined using a method described previously (Mishra et al., 2018b). Various concentrations of Trolox over a range of 400–12.5 µM were taken as standards. Different concentrations (10, 5, 2.5, 1.25 and 1 mg/ml) of all the CSF extracts were prepared. Results were expressed as μM TE/g of extract. Final absorbances were recorded at 593 nm.

### Antibacterial Efficacy of the CSFs

#### Screening of CSF Extracts for Antibacterial Activity

Glycerol stocks of two bacterial strains: *E. coli* and *S. typhi* were revived in nutrient broth for 16–18 h at 37°C, in an incubator-cum-shaker (Orbitek, Chennai, India), at 90 rpm. The pure cultures obtained were streaked on nutrient agar medium. Antibacterial action of CSF1, CSF2 and CSF3 against *E. coli* and *S. typhi* was determined by Kirby Bauer disk diffusion technique (Mishra et al., 2018a). Briefly, 100 µg/ml of each CSF extract dissolved in nutrient broth was applied onto spherical disks made out of Whatman No.1 filter paper. The dried disks were placed on nutrient agar medium plates swabbed with bacterial pathogens under study. Thereafter, these plates were incubated at 37°C for 16–18 h at a shaking speed of 90 rpm. Prominent zones of inhibition formed around the disks were indicative of antibacterial activity. Kanamycin and nutrient broth were taken as the positive and negative controls, respectively.

### Determination of Minimal Inhibitory Concentration

Bacterial colonies approximately, 16–18 h old, cultured on nutrient agar medium were diluted in 0.8% physiological saline to prepare a 0.1 McFarland suspension. The bacterial cultures were inoculated in test tubes containing 5 ml nutrient broth. To these tubes, the specific CSF extracts were added in concentrations over a range of 10–100 µg/ml and incubated at 37°C for 16–18 h at 90 rpm. The lowest concentration of an extract demonstrating inhibition of bacterial growth was determined to be its minimal inhibitory concentration (MIC) for a given bacterial pathogen (Zhang et al., 2015). Kanamycin and pure nutrient broth medium were used as the positive and negative controls, respectively.

### Generation of Reactive Oxygen Species Within Bacterial Cells

Reactive oxygen species (ROS) generation in a bacterial cell upon treatment with a specific CSF extract as a potential antibacterial mechanism was investigated by a method described previously (Zhang et al., 2015). Various concentrations of the CSF samples were added to nutrient broth tubes containing bacterial cells at density of 10^5^ CFU/ml and incubated at 37°C for 3 h. The cultures were then centrifuged at 4°C for 15 min at a speed of 500 × *g*, and the resultant supernatant was treated with 50 µM of 2’,7’-dichlorofluorescein diacetate (DCFDA), for 1 h at 37°C, in dark. A control group untreated with DCFDA was taken as control. The level of ROS generated in the bacterial cultures was recorded in triplicates using a fluorescence spectrophotometer (Cary Eclipse, Santa Clara, CA, United States) at an excitation wavelength of 485 nm and emission wavelength of 528 nm.

### Protein Leakage in Bacterial Cells

Protein leakage instigated within the bacterial pathogen’s cellular environment triggered by particular CSF extracts as yet another antibacterial mechanism was examined (Zhang et al., 2015). Here, 16–18 h old cultures of bacteria in nutrient broth were centrifuged at 10,000 rpm for 20 min, followed by re-suspension in 0.8% physiological saline. After this, the bacterial cells were treated with CSF extracts of varied concentrations. Following an incubation period of 3 h, each bacterial suspension was centrifuged at 12,000 rpm for 15 min and the supernatant was analysed for protein content by Bradford method. Protein concentration at 595 nm was determined using bovine serum albumin (BSA) as standard.

### Inhibition of Efflux Pumps in Bacterial Cells

Bacterial cultures inoculated in nutrient broth were incubated overnight for 24 h at 37°C at 95 rpm. The culture was then centrifuged for 5 min at 4000 rpm. Discarding the supernatant, the pellet collected was suspended in nutrient broth. A 500 µg/ml sample of each CSF extract was prepared in nutrient broth. To individual wells of a 96 well microtiter plate, 150 µl nutrient broth containing bacterial inoculum, 20 µl ethidium bromide (EtBr) and 50 µl of the respective CSF sample was added and incubated for 10 min at room temperature. Thereafter, fluorescence was recorded using a fluorescence spectrophotometer over 30 min with interims of 5 min, at an excitation wavelength of 530 nm and emission wavelength of 600 nm. In this study, 5 µl of carbonyl cyanide 3-chlorophenylhydrazone or, “CCCP” known to be an effective efflux pump inhibitor was taken as the positive control and water served as negative control (Kumar et al., 2016).

### Protective Action of CSF Extracts Against Hypobaric Hypoxia in Human Embryonic Kidney Cell Line

The protective effects of the *C. sinensis* supercritical fluid extract in recuperating human embryonic kidney cells (HEK 293) from hypoxic stress was evaluated by segregating the experimental cells into four groups for every dose (50/75/100/125 µg/ml) of each CSF extract. The groups were: normoxia control (N), normoxia supplemented with CSF extract (N+sample), hypoxia control (H) and, hypoxia supplemented with CSF extract (H+sample). Initially, the cells were cultured in high glucose DMEM (Dulbecco Minimal Essential Medium), (pH 7.2, 37°C, 5% CO_2_) supplemented with antibiotics gentamycin sulphate (100 mg/L) and penicillin (100 mg/L) and enriched with foetal bovine serum (FBS) (10%, v/v) as described in a previous study (Kirar et al., 2017). Cells were grown in 96-well microtitre plates (Nunc, Roskilde, Denmark), maintaining a cell density of 10^5^ cells/cm^2^, and placed in incubator (New Brunswick, Galaxy 170R, Hamburg, Germany) with 21% O_2_ i.e., normoxic condition. Experiments were conducted only on cells that were at least 80% confluent.

For hypoxia stress, cells were cultured for 24 h in a low oxygen environment (0.5% O_2_, 5% CO_2_ and 94.5% N_2_) in New Brunswick Galaxy 48R hypoxia incubator (Hamburg, Germany). Cellular viability of the cells was determined using MTT assay (Mosmann, 1983). Different concentrations (50, 75, 100 and 125 µg/ml) of CSF extracts were supplemented to the growth medium, according to the aforementioned grouping. Dose of each CSF extract demonstrating maximum restoration of cellular viability under hypoxic conditions was inferred as its corresponding optimum dose.

### Western Blotting for Assessing Effect of CSF Extract on HIF-1α Levels

Post hypoxia exposure, cells were de-adhered from the T25 flask by trypsinization (0.1%, v/v in PBS) for about 5 min. Cells were homogenized in radioimmunoprecipitation assay buffer (50 mM/L Tris-HCl, 150 mM/L NaCl, 0.1% sodium dodecyl sulfate, 1% NP-40, 0.5% deoxycholate, and protease inhibitor cocktail 5 µl/ml (MP Biomedicals, Ilkirch, France) and centrifuged at 12,000 × *g* at 4°C for 30 min. The supernatant collected contained cytosolic fractions. Total protein was estimated by Lowry’s method (Nehra et al., 2015). For Western blotting, 25 µg of cytosolic protein was resolved in 10% SDS-PAGE (sodium dodecyl sulphate-polyacrylamide gel electrophoresis) and transferred onto nitrocellulose membrane (Millipore, Bedford, MA, United States) *via* semi-dry trans-blot system (BioRad, Hercules, CA, United States) at 15–20 V for 60 min. Membranes were blocked in blocking buffer composed of 3–5% bovine serum albumin in 0.5% Tris-buffered saline-Tween^®^ (TBST), for 2 h and incubated with primary antibody i.e., polyclonal HIF-1α antibody (1:1000 dilution, E-AB-31662; Elabscience Biotechnology Inc., Houston, TX, United States) for 2 h. After washing in TBST, the membranes were incubated with appropriate secondary antibodies (1:20,000–30,000) (Santa Cruz Biotechnology, Dallas, TX, United States) at room temperature for 1 h, on dancing shaker. HIF-1α being probed was visualized using H_2_O_2_/3,3′,5,5′-tetramethylbenzidine (TMB) (Sigma-Aldrich, St. Louis, MO, United States). Quantitation was done by densitometric analysis using ImageJ software (Kirar et al., 2017).

### Statistical Analysis

Quantitation in all the HPTLC experiments were performed in triplicates. Data from antioxidant, antibacterial and *in vitro* cell culture assays were acquired in triplicate manner for all the CSF extracts in order to determine reproducibility. Results have been expressed as mean ± SD. All the statistical analyses viz., computation of mean and standard deviations were performed using Statistical Package for the Social Sciences (SPSS) software version V 2.21 (IBM, Chicago, IL, United States). One way analysis of variance (ANOVA) was applied to check the level of significance by Bonferroni *post hoc* tests. In all the tests, *p* < 0.05 was taken as a criterion for statistical significance.

For construction of variables factor maps by PCA, data acquisition and processing including generation of covariance matrices and proportion of variances were generated by R, version 4.0.3 (http://cran.r-project.org/).

## Results

**FIGURE 1:**
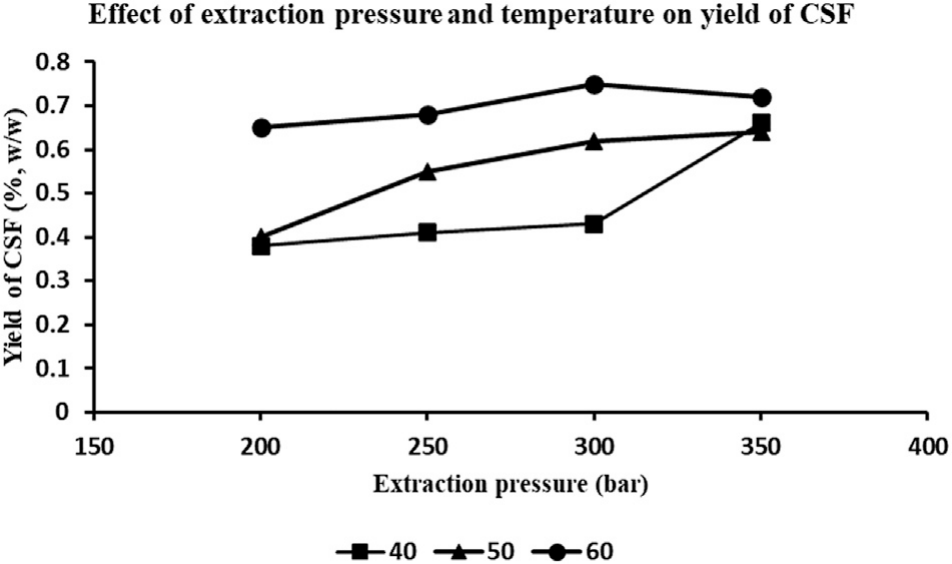
Effect of extraction pressure and temperature on the yields of *C. sinensis* supercritical fluid (CSF) extracts. Combination of various pressures (200, 250, 300 and 350 bar) and temperatures 40, 50, and 60°C were used for the optimization protocol. 1% (v/v) ethanol was used as entrainer. CO_2_ flow rate was 0.4 L/h.

The different combinations of four extraction pressures and three extraction temperatures resulted in acquisition of 12 supercritical CO_2_ extracts, of varying yields. The effect of extraction pressures and temperatures on the yields of extracts is depicted in Figure 1. It is observed that the yields incremented with a combination of rising pressure and temperature, except for 350 bar and 60°C, which displayed lesser yield than that at 300 bar and 60°C. The maximal yields were achieved with combination of *1*) extraction pressure of 300 bar and temperature of 60°C (extract labelled “CSF1”) and, *2*) extraction pressure of 350 bar and temperature of 60°C (extract labelled “CSF2”). Keeping the former condition intact but without the use of entrainer wielded the third extract “CSF3.” These three *C. sinensis* supercritical CO_2_ extracts, were collectively referred to as “CSF extracts.” The yields were 0.75, 0.72 and 0.53% (w/w), for CSF1, CSF2 and CSF3, correspondingly. The physical properties of all the *C. sinensis* supercritical extracts were viewed to be sticky in nature and dark brownish in colour.

### HPTLC Characterization Confirmed the Presence of Cordycepin, Flavonoids and Nucleobases in the CSF Extracts

**FIGURE 2:**
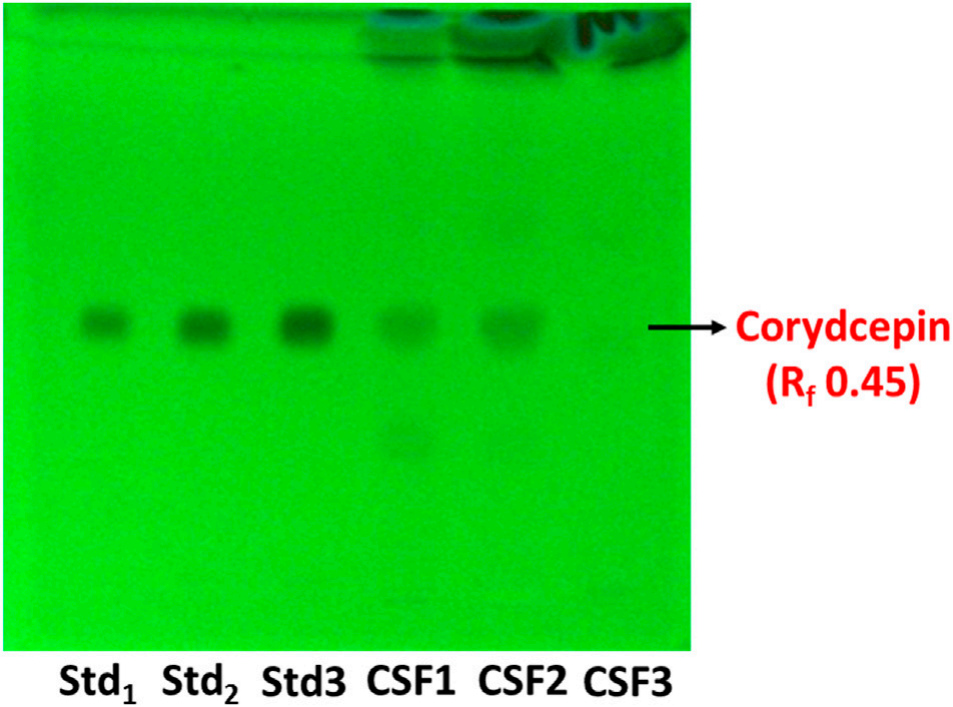
HPTLC chromatogram at 254 nm shows bands distinctive of cordycepin, the main marker metabolite used for quality assessment of *Cordyceps* spp. The retardation factor (R_f_) was determined to be 0.45. Lanes labelled Std_1_, Std_2_ and Std_3_ contain 0.25, 0.5 and 1 µg of standard cordycepin.

HPTLC fingerprinting given in Figure 2 illustrated distinct bands indicative of cordycepin, the main marker metabolite of *C. sinensis* in CSF1 and CSF2, but not in CSF3. Cordycepin was quantitated to be fairly more in CSF1 with 296.6 ± 15.3 µg/mg of extract than CSF2 (267.1 ± 9.2 µg/mg of extract).

**FIGURE 3:**
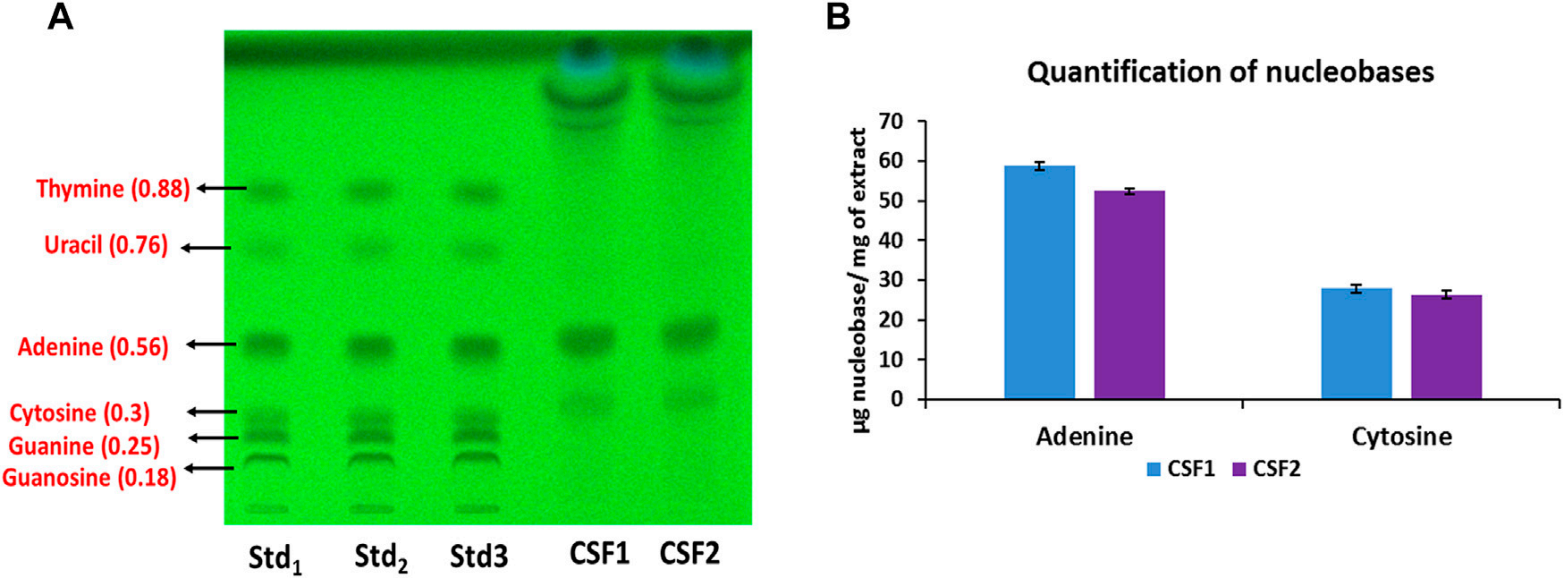
HPTLC chromatogram at 254 nm confirms the presence of adenine and cytosine in CSF1 and CSF2 extracts. Respective retardation factor values are written in parentheses **(A)**. Quantities detected from densitometric scanning are also given **(B)**. Lanes labelled Std_1_, Std_2_ and Std_3_ contain 0.5, 0.58 and 0.66 µg of each standard nucleobase. Values for quantification are represented as mean ± SD (*n* = 3).

Figure 3A represents the HPTLC chromatogram for identification of nucleobases in *C. sinensis* supercritical extracts. Among the nucleobases under consideration, adenine and cytosine were detected in CSF1 and CSF2. Figure 3B denotes the quantities of these nucleobases as calculated from densitometric scanning. It was perceived that CSF1 consisted of a marginally higher content of adenine (58.85 ± 0.24 µg/mg of extract) and cytosine (27.94 ± 1.81 µg/mg of extract) than that in CSF2, where adenine content was 52.43 ± 0.68 µg/mg of extract and cytosine was 26.35 ± 1.07 µg/mg of extract.

**FIGURE 4:**
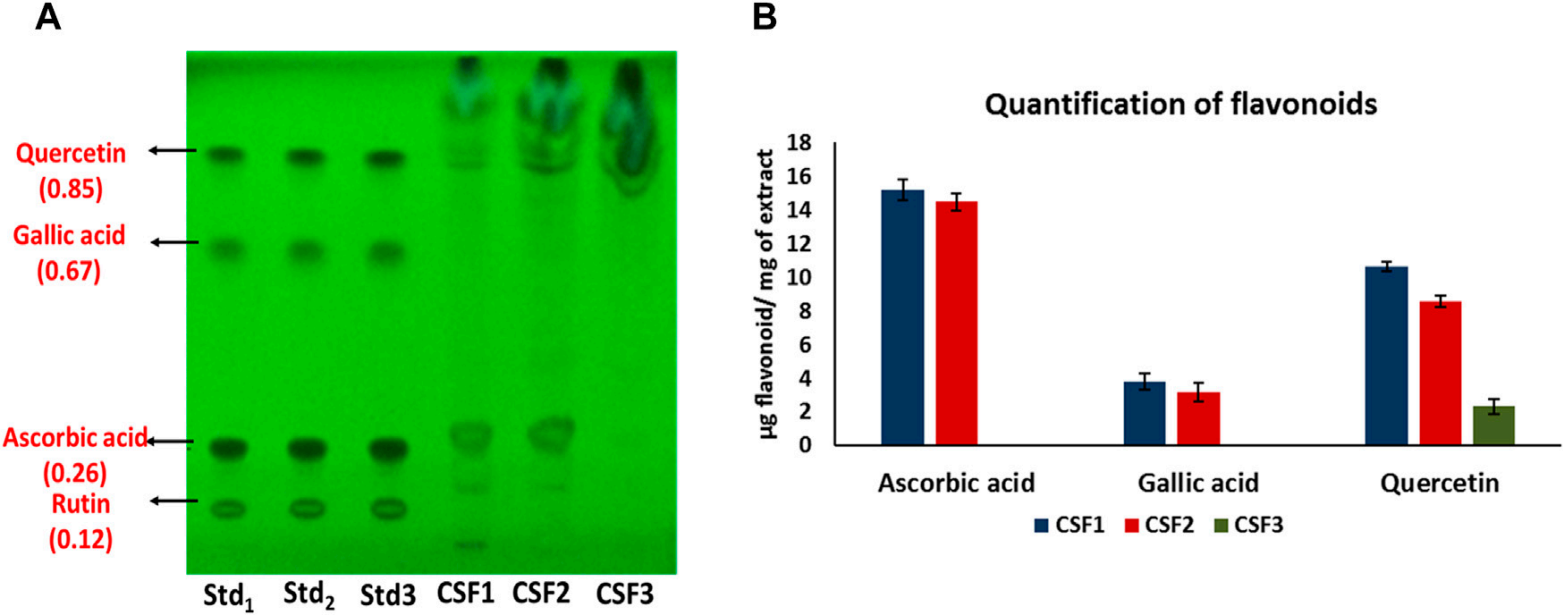
HPTLC chromatogram at 254 nm confirms the presence of ascorbic acid, gallic acid and quercetin in CSF1 and CSF2 extracts, and only quercetin in CSF3 extract. Respective retardation factor values are written in parentheses **(A)**. Quantities detected from densitometric scanning are also given **(B)**. Lanes labelled Std_1_, Std_2_ and Std_3_ contain 0.75, 0.875 and 1 µg of each standard flavonoid. Values for quantification are represented as mean ± SD (*n* = 3).

Figure 4A represents the HPTLC profiling for flavonoids detected in *C. sinensis* supercritical extracts. Here, presence of ascorbic acid, gallic acid and quercetin was confirmed in CSF1 and CSF2. Figure 4B denotes the quantities of these flavonoids as measured using densitometric scanning. It was clearly seen that CSF1 possessed the highest quantity of all three aforesaid flavonoids having ascorbic acid, gallic acid and quercetin in amounts of 15.2 ± 0.6, 3.8 ± 0.48 and 10.63 ± 0.26 µg/mg of extract. Corresponding values of CSF2 were 14.48 ± 0.5, 3.18 ± 0.19 and 8.55 ± 0.28 µg/mg of extract. CSF3 was found to have only quercetin (2.31 ± 0.27 µg/mg of extract). The identification and quantification of bands corresponding to standard nucleobases or flavonoids has been performed within the permissible evaluation window of winCATS software.

### Presence of Putative Bioactive VOCs Verified by GC-MS Analysis

**FIGURE 5:**
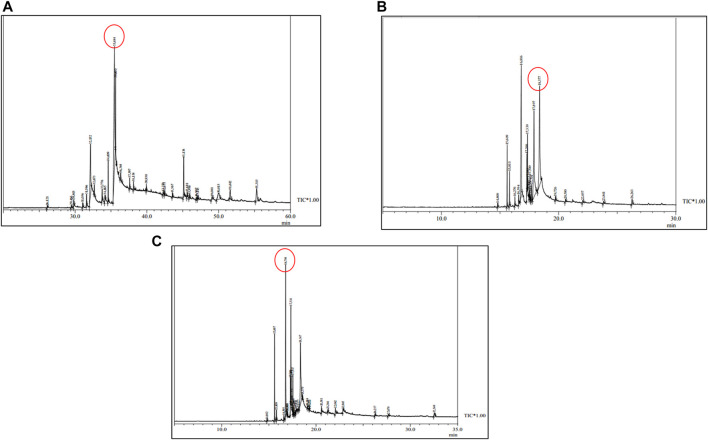
Total ion chromatograms obtained after GC-MS analysis of *Cordyceps sinensis* supercritical carbon dioxide extracts. Based on area percentages, the most abundant volatile organic compounds in CSF1, CSF2 and CSF3 were revealed to be 9,12-octadecadienoic acid (Z,Z)- **(A)**; 9,12-octadecadienoic acid (Z,Z)-, trimethylsilyl ester **(B)** and hexadecanoic acid, trimethylsilyl ester **(C)**, respectively.

The CSF extracts were observed to comprise various VOCs inclusive of alcohols, esters, stearates, terpenes, etc. The major VOCs detected in the supercritical CO_2_ extracts with their respective retention times, peak areas and area percentages are depicted in Tables 1–3 for CSF1, CSF2 and CSF3, respectively. Results based on the area percentages of individual VOCs established that 9,12-octadecadienoic acid (Z,Z)- or, α-linoleic acid was the most abundant VOC in CSF1; 9,12-octadecadienoic acid (Z,Z)-, trimethylsilylester i.e., linoleic acid trimethylsilyl ester was the most abundant VOC in CSF2 whereas hexadecanoic acid, trimethylsilyl ester (palmitic acid TMS) was the most abundant VOC in CSF3. Total ion chromatograms of the aforesaid most abundant VOCs are represented in Figure 5. All the three supercritical extracts individually had certain VOCs which are unique to each of them, thus proving that every individual sample had its own distinct characteristics in terms of bioactivity, as discoursed later.

**TABLE 1:** List of major volatile organic compounds detected in CSF1 extract by GC-MS.

Peak	Retention time (R_t_), min	Area	Area%	Name
1	26.121	419,928	0.29	1-TETRADECANOL, ACRYLATE
2	29.466	444,072	0.30	TETRADECANOIC ACID, TRIMETHYLSILYL ESTER
3	29.803	1,550,751	1.07	PENTADECANOIC ACID
4	31.056	858,623	0.59	HEXADECANOIC ACID, METHYL ESTER
5	31.596	2,185,882	1.50	*n*-PENTADECANOIC ACID, TRIMETHYLSILYL ESTER
6	32.152	20,965,594	14.40	PENTADECANOIC ACID
7	32.673	1,888,914	1.30	HEXADECANOIC ACID, ETHYL ESTER
8	33.776	2,952,332	2.03	HEXADECANOIC ACID, TRIMETHYLSILYL ESTER
9	34.165	1,824,254	1.25	9-OCTADECENOIC ACID (Z-)
10	34.609	6,324,673	4.34	9,12-OCTADECADIENOIC ACID (Z,Z)-, METHYL ESTER
11	35.484	56,200,296	38.60	9,12-OCTADECADIENOIC ACID (Z,Z)-
12	35.650	10,256,586	7.04	ETHYL (9Z,12Z)-9,12-OCTADECADIENOATE #
13	36.364	2,484,453	1.71	LINOSAEURE, TRIMETHYLSILYL ESTER
14	37.597	1,243,151	0.85	OCTANAMIDE, N-(2-HYDROXYETHYL)-
15	38.138	1,785,300	1.23	METHYL OCTADECA-9,12-DIENOATE
16	39.930	759,375	0.52	1,2-BENZENEDICARBOXYLIC ACID
17	42.270	679,937	0.47	13-DOCOSENAMIDE, (Z)-
18	42.475	592,267	0.41	DECANEDIOIC ACID, BIS (2-ETHYLHEXYL) ESTER
19	43.567	790,657	0.54	TETRACONTANE
20	45.138	9,853,510	6.77	9(11)-DEHYDROERGOSTERYL BENZOATE
21	45.618	1,157,108	0.79	9(11)-DEHYDROERGOSTERYL BENZOATE
22	45.936	1,053,723	0.72	HEXADECANOIC ACID, METHYL ESTER
23	46.907	537,691	0.37	TETRACONTANE
24	47.115	717,398	0.49	PYRAZINE, TETRAKIS (1-METHYLETHYL)-
25	49.081	1,162,320	0.80	ERGOSTA-5,7,9(11),22-TETRAEN-3-OL, (3.BETA.,22E)
26	50.015	6,610,013	4.54	ERGOSTEROL
27	51.612	3,491,024	2.40	16-HENTRIACONTANONE
28	55.315	6813,607	4.68	10,13-DIMETHYL-17-(1,4,5-TRIMETHYL-HEX-2-ENYL
		145,603,439	100	

The retention times, peak areas and area percentages are indicated.

**TABLE 2:** List of major volatile organic compounds detected in CSF2 extract by GC-MS.

Peak	Retention time (R_t_), min	Area	Area%	Name
1	14.800	145,752	0.36	TETRADECANOIC ACID, TRIMETHYLSILYL ESTER
2	15.609	2,388,227	5.84	HEXADECANOIC ACID, METHYL ESTER
3	15.811	1,402,036	3.43	*n*-PENTADECANOIC ACID, TRIMETHYLSILYL ESTER
4	16.270	322,754	0.79	HEXADECANOIC ACID, ETHYL ESTER
5	16.594	312,992	0.76	*cis*-9-HEXADECENOIC ACID, TRIMETHYLSILYL ESTER
6	16.816	9,859,447	24.09	HEXADECANOIC ACID, TRIMETHYLSILYL ESTER
7	17.290	1,431,582	3.50	9,12-OCTADECADIENOIC ACID (Z,Z)-, METHYL ESTER
8	17.339	1,803,748	4.41	9-OCTADECENOIC ACID, METHYL ESTER
9	17.383	353,836	0.86	9-OCTADECENOIC ACID (Z-), METHYL ESTER
10	17.500	253,523	0.62	9,12-OCTADECADIENOIC ACID (Z,Z)-, TRIMETHYLSILYL ESTER
11	17.549	765,219	1.87	METHYL STEARATE
12	17.611	176,041	0.43	METHYL 15-HYDROXY-9,12-OCTADECADIENOATE
13	17.701	933,283	2.28	HEPTADECANOIC ACID, TRIMETHYLSILYLESTER
14	17.897	9,004,280	22.00	9,12-OCTADECADIENOIC ACID (Z,Z)-
15	18.377	10,633,336	25.99	9,12-OCTADECADIENOIC ACID (Z,Z)-, TRIMETHYLSILYL ESTER
16	19.726	192,583	0.47	CYCLOPROPANEOCTANOIC ACID,2-[(2-ETHYLCYCLOPROPYL)METHYL ESTER]
17	20.580	134,002	0.33	1,3,5-TRISILACYCLOHEXANE
18	22.057	194,423	0.48	1,3-DIOXOLANE, 2-METHOXY-4-HEXADECENYL ESTER
19	23.841	150,386	0.37	SQUALENE
20	26.263	462,562	1.13	9(11)-DEHYDROERGOSTERYL BENZOATE
		409,200,002	100	

The retention times, peak areas and area percentages are indicated.

**TABLE 3:** List of major volatile organic compounds detected in CSF3 extract by GC-MS.

Peak	Retention time (R_t_), min	Area	Area%	Name
1	14.802	28,836	0.21	TETRADECANOIC ACID, TRIMETHYLSILYL ESTER
2	15.607	1,519,042	11.10	HEXADECANOIC ACID, METHYL ESTER
3	15.809	192,622	1.41	*n*-PENTADECANOIC ACID, TRIMETHYLSILYL ESTER
4	16.595	75,701	0.55	OCTADECANOIC ACID, METHYL ESTER
5	16.784	3,575,513	26.14	HEXADECANOIC ACID, TRIMETHYLSILYL ESTER
6	16.924	45,600	0.33	ANDROST-1-EN-3-ONE, 17-HYDROXY (5.α., 17.β.)-
7	16.980	157,023	1.15	17-OCTADECEN-14-YNOIC ACID, METHYL ESTER
8	17.284	463,156	3.39	9,12-OCTADECADIENOIC ACID (Z,Z)-, METHYL ESTER
9	17.334	1,503,058	10.99	9-OCTADECENOIC ACID, METHYL ESTER
10	17.380	240,987	1.76	9-OCTADECENOIC ACID (Z-), METHYL ESTER
11	17.436	51,640	0.38	METHYL OCTADECA-9,12-DIENOATE
12	17.547	647,472	4.73	METHYL STEARATE
13	17.608	142,648	1.04	METHYL 15-HYDROXY-9,12-OCTADECADIENOATE
14	17.697	210,047	1.54	HEPTADECANOIC ACID, TRIMETHYLSILYL ESTER
15	17.813	60,642	0.44	4-BROMOBUTANOIC ACID, DODEC-3-YNYL ESTER
16	17.891	78,198	0.57	OXACYCLOHEPTADEC-8-EN-2-ONE, (8Z)
17	18.127	65,294	0.48	9,12-OCTADECADIENOIC ACID, METHYL ESTER
18	18.347	3,477,306	25.42	9,12-OCTADECADIENOIC ACID (Z,Z)-, TRIMETHYLSILYL ESTER
19	18.570	102,810	0.75	OCTADECANOIC ACID, TRIMETHYLSILYL ESTER
20	19.094	30,316	0.22	DOCOSANOIC ANHYDRIDE
21	19.272	28,802	0.21	2, PYRROLIDINONE, 1-[2-(4-PIPERIDINYL)ETHYL]-
22	19.322	24,106	0.18	EICOSANOIC ACID, METHYL ESTER
23	20.582	149,004	1.09	1,3,5-TRISILACYCLOHEXANE
24	21.266	59,380	0.43	HEXADECANOIC ACID, 4-TRIMETHYLSILYL ESTER
25	22.062	249,823	1.83	5,5-DIMETHYL-1,3-DIOXANE-2-ETHANOL, TERT-BUTYLDIMETHYLILYL ESTER
26	22.865	95,494	0.70	9-OCTADECENOIC ACID (Z)-,2-TRIMETHYLSILYL ESTER
27	26.257	851,928	0.62	9(11)-DEHYDROERGOSTERYL BENZOATE
28	27.678	91,601	0.67	STIGMASTA-4,7,22-TRIEN-3.BETA.-OL
29	32.549	229,002	1.67	16-HENTRIACONTANONE
		13,680,321	100	

The retention times, peak areas and area percentages are indicated.

### Metabolomics by PCA Based on Chromatographic Data Verified Inter-relatedness Between CSF1 and CSF2

The chromatographic data acquired from metabolite profiling were subjected to chemometric analysis in an open source R studio, to carry out data reduction and pattern recognition. In case of both HPTLC and GC-MS data, two major principal components, represented as Dim1 and Dim2 depicted the overall variance over the entire dataset. The primary axis, i.e., the first principal component describes majority of the variance in a factor map.

**FIGURE 6:**
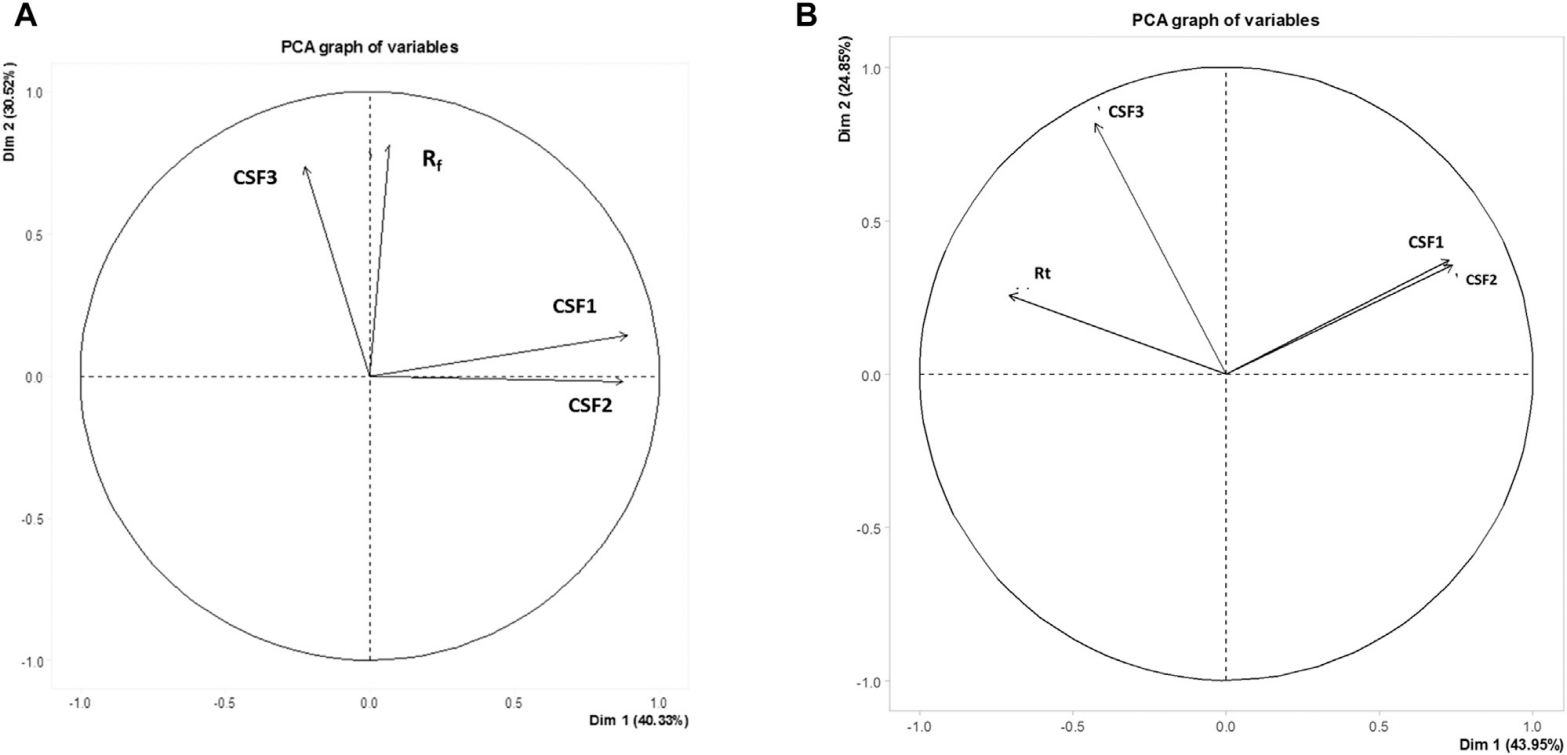
Variables factor map depicting variances among CSF1, CSF2 and CSF3 extracts on basis of HPTLC **(A)** and GC-MS **(B)** metabolite profiling. Similarities were observed between CSF1 and CSF2, the supercritical extracts prepared using ethanol as co-solvent, owing to their close presence in same quadrant. CSF3 occurred on a separate quadrant from the former. R_f_, retardation factor value of metabolites detected by HPTLC; R_t_, retention time of VOCs identified by GC-MS.

For HPTLC data, all the retardation factors from analysis of both nucleobases as well as flavonoids were taken into account to form data matrix. Information pertaining to retardation factor values and peak areas of 98 metabolites were compressed to manifest the entire data in form of Dim 1 and Dim 2, whose values were 40.33 and 27.91% in variables factor map (Figure 6A). Data compression in PCA ensures that there is no loss in variances while describing the dataset.

Similarly, the factor map constructed on basis of retention times of 67 constituent VOCs exhibited a variance of Dim 1 = 43.95% and Dim 2 = 24.85% (Figure 6B).

It can be clearly seen that CSF3 was present in separate quadrants, distinctive from CSF1 and CSF2, in case of both HPTLC as well as GC-MS profiling. This can be linked to the difference in metabolome owing to variation in extract preparation protocol. Moreover, the presence of CSF1 and CSF2 in the same quadrant and their proximity to each other illustrated their obvious interrelatedness (Figure 6).

### CSF Extracts Exhibited Substantial Free Radical Scavenging and FRAP Activities

**FIGURE 7:**
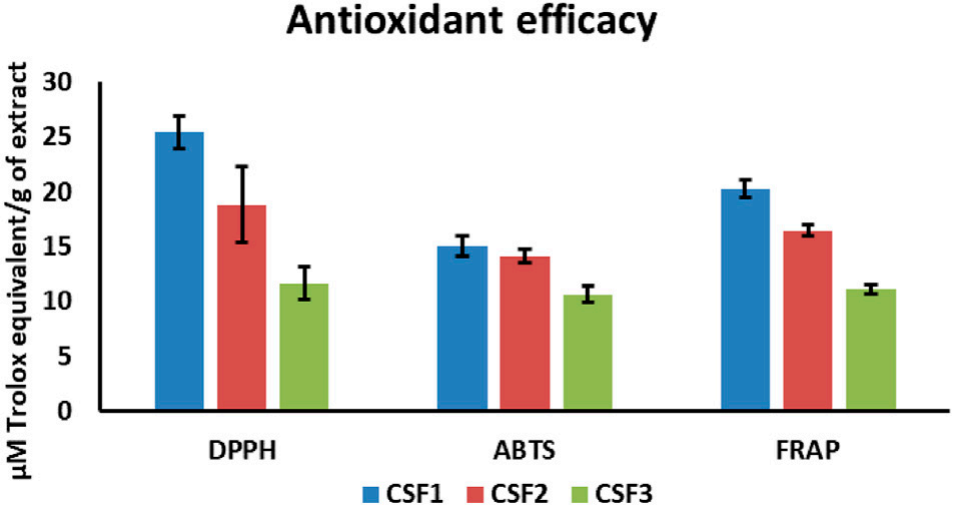
Among all the supercritical CO_2_ extracts of *Cordyceps sinensis*, CSF1 was discerned to have the highest *in vitro* free radical (DPPH, ABTS) scavenging activity and ferric ion reducing power (FRAP). Values are represented as mean ± SD (*n* = 3).

All the CSF extracts were capable of antioxidant power by virtue of free radical scavenging as well as ferric ion reduction. DPPH radical scavenging activities were 25.39 ± 1.48, 18.82 ± 3.42, and 11.65 ± 1.44 μM TE/g of extract, in that order for CSF1, CSF2 and CSF3. Similarly, ABTS assays brought out an activity of 15.05 ± 0.91, 14.12 ± 0.65, and 10.64 ± 0.72 μM TE/g of extract, correspondingly for CSF1, CSF2 and CSF3. FRAP results of the three supercritical CO_2_ extracts explicated the antioxidant potential as 20.22 ± 0.82, 16.47 ± 0.44 and 11.14 ± 0.43 μM TE/g of extract. Overall, it can be verified from above results that CSF1 had the highest antioxidant potency over the rest supercritical fluid extracts (Figure 7).

### CSF Extracts Were Observed to Be Potent Antibacterial Leads Against *E. coli* and *S. typhi*

The significant presence of bioactive metabolites in the CSF extracts and their effective antioxidant abilities stimulated exploration of further bioeffects. In this context, evaluation of antibacterial activities clearly exhibited the efficacy of all three CSF extracts as potential antibacterial agents against pathogenic strains of *E. coli* and *S. typhi*.

**FIGURE 8:**
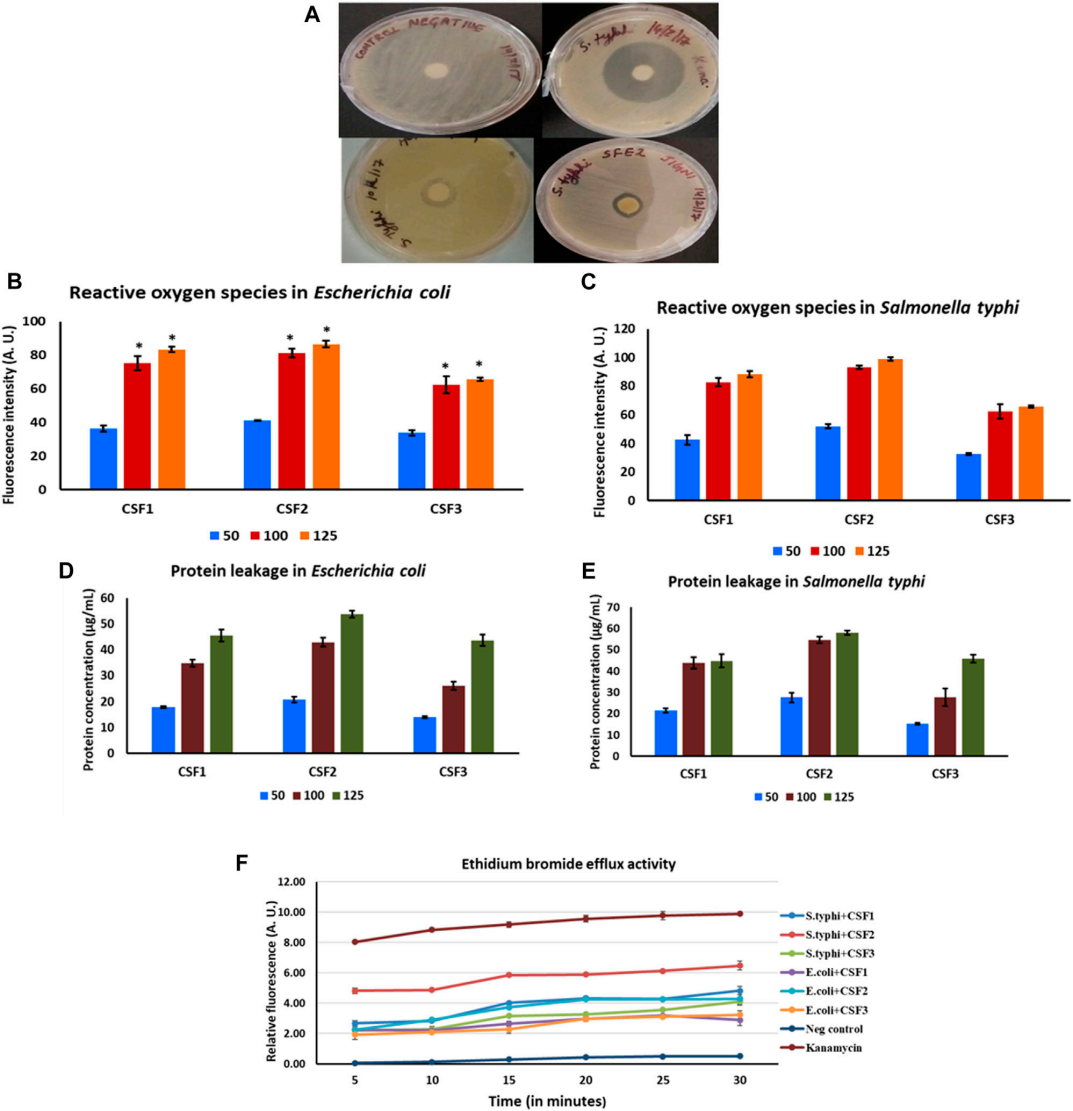
Distinct zones of inhibition after treatment with CSF1 and CSF2 extracts, against pathogenic *Salmonella typhi* can be clearly seen **(A)**. Generation of reactive oxygen species after treatment with different doses (50, 100, 125 µg/ml) of *Cordyceps sinensis* supercritical fluid extracts within *E. coli* **(B)** and *S. typhi* **(C)** was deduced as a potential antibacterial mode of action. Induction of protein leakage of aforesaid extracts against *E. coli* **(D)** and *S. typhi* **(E)** was confirmed to be yet another inhibitory mechanism. The supercritical fluid extracts also led to inhibition of efflux pump activities in the bacterial cells **(F)**. Nutrient broth and kanamycin acted as negative and positive controls, respectively. Data are expressed as mean ± SD (*n* = 3). * represents significant change at level of *p* < 0.05.

For CSF1, agar disc diffusion studies brought out distinct zones of inhibition of diameters 7.0 ± 0.5 mm and 8.5 ± 0.7 mm against *E. coli* and *S. typhi*. Likewise, CSF2 wielded zones of inhibition of 7.5 ± 0.7 mm and 8.2 ± 1.0 mm averting *E. coli* and *S. typhi.* For these two pathogens, CSF3 exerted comparatively lesser antibacterial action wherein the zones of inhibition were 6.5 ± 0.5 mm and 6.5 ± 1.5 mm. The positive control, kanamycin gave distinct zones of inhibition of 30 ± 2 mm against *E. coli* and 35 ± 1.5 mm against *S. typhi*. The zones of inhibition and MIC values of all CSF extracts are noted in Table 4. Representative figure illustrating discrete zones of inhibition of CSF1 and CSF2 against *S. typhi* is given in Figure 8A.

**TABLE 4:** Inhibitory action of *Cordyceps sinensis* supercritical carbon dioxide extracts on bacterial pathogens, *Escherichia coli* and *Salmonella typhi*.

Extract	Zone of inhibition (mm)	MIC (µg/ml)
CSF1:		
*E. coli*	7.0 ± 0.5	50
*S. typhi*	8.5 ± 0.7	35
CSF2:		
*E. coli*	7.5 ± 0.7	40
*S. typhi*	8.2 ± 1.0	45
CSF3:		
*E. coli*	6.5 ± 0.5	60
*S. typhi*	6.5 ± 1.5	65
Kanamycin:		
*E. coli*	30 ± 2	5
*S. typhi*	35 ± 1.5	5

Values for diameters of zones of inhibition (in mm) are given as mean ± SD (*n* = 3). Values in µg/ml indicate the specific minimal inhibitory concentrations (MIC). Kanamycin antibiotic was taken as the positive control against both pathogenic strains.

Owing to promising insights of CSF extracts being prospective antibacterials, certain typical mechanisms of inhibitory action were examined. Generation of ROS, induction of protein leakage and inhibition of efflux pumps were confirmed to be the modes of antibacterial action.

ROS generated in bacterial microenvironment after exposure to CSF extracts were measured using DCFDA. A linearly upward trend was indicated in levels of ROS formed with increasing doses of CSF1, CSF2 and CSF3 over 50–125 µg/ml, against both *E. coli* (Figure 8B) and *S. typhi* (Figure 8C). The bactericidal action was also analysed by probing concomitant leakage of intracellular proteins upon treatment with the CSF extracts. Results portrayed that incremented doses of these extracts resulted in higher amount of protein leakage from the bacterial pathogens–*E. coli* as seen from Figure 8D and *S. typhi* (Figure 8E). This observation was consonant with those obtained for ROS generation that the extent of bactericidal action was proportionate to CSF extract doses. Figures 8B–E also depict that CSF2 showed the most potent abilities in generation of ROS and eliciting protein leakage in the bacterial pathogens.

Besides, it was also proven that CSF2 followed by CSF1 and finally, CSF3 effectually displayed EtBr efflux pump inhibitory activity against *E. coli* and *S. typhi*, thus revealing yet another essential mode of bactericidal action. The results are indicated in Figure 8F.

### CSF Extracts Conferred Protective Action in HEK 293 Cells Against Hypobaric Hypoxia

Previous literature has sufficient evidence to testimony that hypoxic stress noticeably damages the normal functioning of kidney, thus leading to renal failure (Fu et al., 2016). Thus, it is imperative to develop therapeutics for recuperating the detrimental effects of hypobaric hypoxia. With this perspective, the proficiency of *C. sinensis* supercritical CO_2_ extracts as protective leads against hypoxia in human embryonic kidney, HEK 293 cells was probed.

**FIGURE 9:**
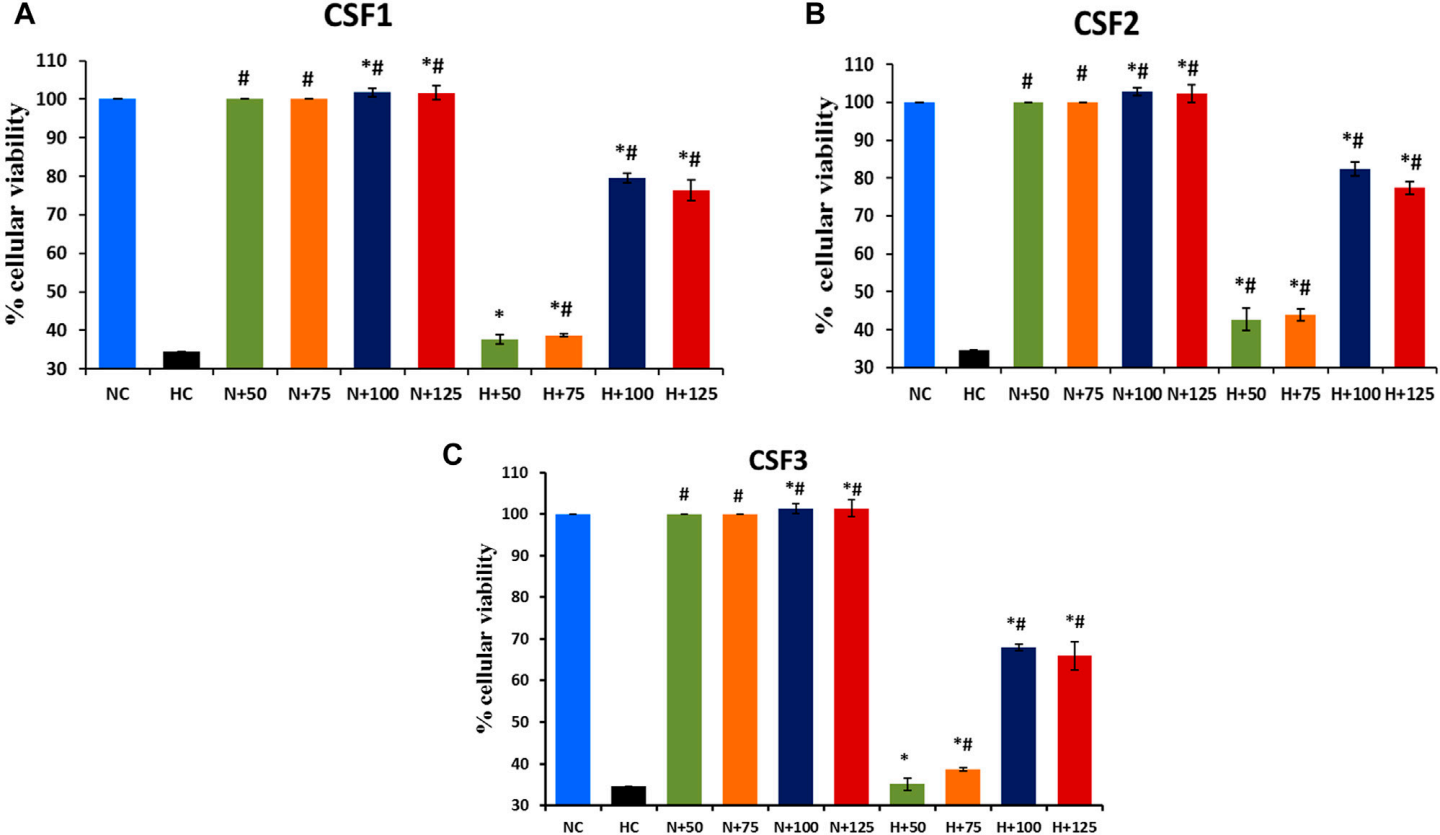
Restoration of cellular viability by *C. sinensis* supercritical CO_2_ extracts. At an optimum dose of 100 µg/ml, CSF1, CSF2 and CSF3 improved HEK 293 cellular viability to 79.53% **(A)**, 82.36% **(B)** and 68% **(C)**, under hypoxia. N, normoxia; H, hypoxia; N+x, supplementation of various doses (x = 50/75/100/125 µg/ml) of CSF in normoxia; H+x, supplementation of various doses (x = 50/75/100/125 µg/ml) of CSF in hypoxia. Results are expressed as mean ± SD (*n* = 3). * represents significant change at level of *p* < 0.05 with respect to N, # represents significant change at level of *p* < 0.05 with respect to H.

Exposure to hypoxia in culture conditions significantly reduced the viability of HEK 293 cells to 37.5%, in contrast to 100% cell viability under normoxia. Significant restoration (*p* < 0.05) of cell viability under hypoxic milieu was brought about by the addition of CSF extracts. The effect of *C. sinensis* supercritical extracts in efficaciously countering the debilitating impact of hypoxia milieu is shown in Figure 9. Out of all the different doses that is, 50/75/100/125 µg/ml of the subject extracts being studied, 100 µg/ml was ascertained to be the optimal dose. The best cytoprotective action against hypoxia was conferred by CSF2 whose optimum dose of 100 µg/ml revived cell viability to 82.36 ± 1.76% (Figure 9B). This was followed by CSF1 and then CSF3 that improved cellular viability to 79.53 ± 1.33% (Figure 9A) and 68 ± 0.75% (Figure 9C), in that order.

### CSF2 Efficiently Modulated HIF-1α in HEK-293 Cells, as a Predominant Protective Mechanism Against Hypobaric Hypoxia

**FIGURE 10:**
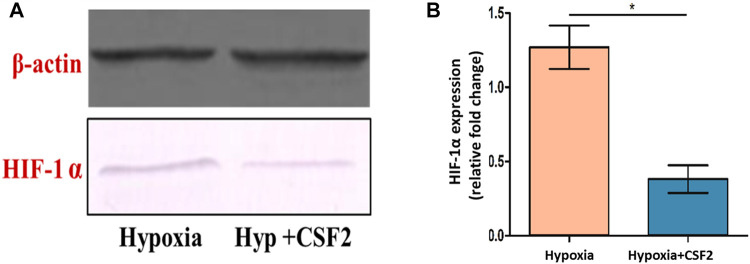
Western blot showing level of hypoxia inducible factor-1 alpha (HIF-1α) (**A**), which otherwise elevated under hypoxia stress, was decreased after supplementation with CSF2 (**B**). Hypoxia+CSF2 indicates supplementation of 100 µg/ml of CSF2 extract in HEK 293 cells being cultured in low oxygen tension (0.5% O_2_). Results are expressed as mean ± SD (*n* = 3). * represents significant change at level *p* < 0.05.

The effect of the CSF extract showing highest protective potential, i.e, CSF2 was studied on modulation of HIF-1α, the main marker of cell survivability under low oxygen tension. Western blotting experiments brought out that level of HIF-1α, which was otherwise heightened in hypoxia conditions, was visibly diminished after supplementation with CSF2. β-Actin served as the loading control (Figure 10A). Densitometry by ImageJ revealed that CSF2 influenced a four-fold downregulation in the levels of HIF-1α, as verified from Figure 10B.

## Discussion

The use of supercritical fluids is gaining ground as a “green technology” for faster production of bioactive extracts of higher purity, with the use of non-toxic solvents like water or CO_2_. It is worth mentioning here that water has a critical temperature of 374°C and critical pressure of 22.1 MPa whereas CO_2_ has parallel values of 31.2°C and 7.3 MPa. These moderate operational requirements of CO_2_ make it a preferred choice over water in supercritical fluid extraction. The major advantages of using supercritical CO_2_ from a pharmacological viewpoint is that, since the process parameters i.e., supercritical conditions are moderate, there is no risk of extract degradation. In addition, by bringing about slight variations in temperature or pressure, the selectivity of target compound can be varied. Moreover, CO_2_ as a solvent is categorized as GRAS (“generally regarded as safe”), hence end products are not harmful or toxic. Also, the products are almost residue free; even if co-solvents like ethanol are added, they are easily removed (Bhusnure et al., 2015). Also, carbon dioxide has low viscosity and better diffusivity capacity in solid matrices owing to its dual gaseous and liquid-like properties, thus efficiently solubilizing the desired bioactive compounds (Capuzzo et al., 2013). Supercritical CO_2_ has been reportedly used for separation of alkaloids, carotenoids, essential oils, flavones, preparation of several value-added pharmaceuticals, cosmetics, nutraceuticals, food additives, etc. (Machmudah et al., 2006; Takeuchi et al., 2010; Rahimi et al., 2011; Kagliwal et al., 2012).

The only shortcoming linked to the usage of supercritical CO_2_ is its low polarity index that obstructs efficient extraction of polar compounds. This limitation is overcome with the addition of entrainers or co-solvents like methanol, ethanol, or water. Use of co-solvents indirectly enhances the solute contact with solvent, thus, offering an augmented overall solubility and optimum mass transfer, especially of bioactive polar compounds in the extract (Walsh et al., 1987; Pronyk and Mazza, 2009). Previous reports cite that the usage of co-solvents result in efficient extraction of polar compounds (Antunes-Ricardo et al., 2017; Wrona et al., 2017). It is always ensured that the co-solvent is added temperately, mostly ranging from 1 to 5% (w/w) so as to minimize time taken for its removal or recovery, *per se*. Ethanol being a food grade modifier is often used as the co-solvent for choice while preparing supercritical extracts for pharmacological or nutraceutical purposes (Sovová et al., 2001; Macías-Sánchez et al., 2009; Capuzzo et al., 2013; Nagavekar and Singhal., 2018) including separation of bioactive compounds from various traditional Chinese medicinal sources (Li, 2007).In this outlook, supercritical CO_2_ along with ethanol as an entrainer was used for preparing supercritical extracts from the Indian high-altitude variety of “keera jhari” or *C. sinensis*.

Previous studies have established that an increase in extraction pressure enhances the fluid density, thus, increasing the solvating power, hence resulting in higher extraction yield. However, the effect of temperature in supercritical fluid extraction is not always linear. The influence of temperature on extraction is a lot more difficult to predict than that of pressure due to the above stated counter effects. An increase in temperature might lead to increase or decrease in yield, or even not affect the yield at all, depending on the competing effect between fluid density and solute vapor pressure (Shao et al., 2014; Danlami et al., 2015; Hamid et al., 2018).

The results in our study were in agreement with the aforesaid observations where the higher yields were obtained with the highest extraction pressures, i.e., 300 and 350 bar. Also, the yields incremented with a combination of rising pressure and temperature, except for 350 bar and 60°C, which displayed lesser yield than that at 300 bar and 60°C (Figure 1). Since the major objective of our study was to establish the bioefficacy of the *C. sinensis* supercritical CO_2_ extracts rather than an elaborate response-based analysis, we selected only those extracts of maximal yields, i.e., CSF1 (300 bar, 60°C) and CSF2 (350 bar, 60°C) for further analyses. For preparation of CSF3, i.e., without entrainer, the best yielding combination of pressure of 300 bar and temperature of 60°C was maintained.

With respect to the flow rate, certain studies have reported that higher solvent flow rate raises the operational and capital costs. Moreover, efficiency of the column is compromised with increased CO_2_ flow rate, since the HTU (height of a transfer unit) increases with increasing CO_2_ loading. Also, a high flow rate may cause decrease in the yield either by increasing analyte loss during fluid decompression or by increasing the pressure drop across the extraction cell. On the other hand, a very low flow rate might not facilitate ideal extraction at all (Mendiola et al., 2013; Shao et al., 2014). Keeping these factors in consideration, a moderate flow rate of 0.4 L/h was maintained in this study.

The three final extracts - CSF1, CSF2 and CSF3 had yields 0.75, 0.72 and 0.53% (w/w), respectively. Akanda et al. (2012) have cited that pressure, temperature and time can influence the supercritical extraction procedure, in turn, affecting the yields. The manner in which the above parameters affect the actual extraction depends on the type of the raw material and also on the target metabolite. In the present study, the extraction temperature was kept constant at 60°C. A pressure of 300 bar was applied over 2 h for CSF1. A higher pressure i.e., 350 bar was applied for a shorter time to obtain CSF2. The better yield of CSF1 than CSF2, despite a marginally higher extraction pressure, can be ascribed to the longer extraction time in the former. The noticeably lower yield of CSF3 highlighted the significant procedural impact of ethanol as a co-solvent, which at a volume as low as 1%, enhanced the overall yield, and also the content of metabolites in CSF1 and CSF2, as discussed in subsequent paragraphs. The yields in current study were lower than conventional extraction methods wherein the yield of aqueous extract obtained by hot water extraction was reportedly 25.4% (Zheng et al., 2008). The yield as observed in case of accelerated solvent extraction was 33.3% (Rakhee et al., 2016). Despite lower yields, the supercritical CO_2_ extracts prepared here were rich in bioactive metabolites as discussed in following paragraphs.

The supercritical extracts were quantitatively characterized by HPTLC in terms of cordycepin, nucleobases and flavonoids. High throughput, ease of workflow and efficient automation in HPTLC procedure makes it an ideal tool for identification and quantification of bioactive molecules in natural extracts (El-Gindy et al., 2001). Cordycepin or 3’-deoxyadenosine, the predominant functional marker of *Cordyceps* spp. was quantified by HPTLC for quality assessment of the CSFs. It was found that CSF1 and CSF2 exhibited distinct bands suggestive of cordycepin, at quantities 296.6 ± 15.3 µg/g extract and 267.1 ± 9.2 µg/g extract, respectively. As depicted in Figure 1, there was a directly proportionate effect of extraction pressure on the extract yield, except the decrease at 350 bar, 60°C. Thus, CSF1 had better yield than CSF2. This lesser overall yield of CSF2 than CSF1 possibly influenced the lesser amount of cordycepin in CSF2, in comparison to CSF1. In addition, the longer extraction time period also contributed to the higher quantity of cordycepin detected in CSF1. CSF3, whose preparation was bereft of ethanol as co-solvent did not display any band specific to cordycepin, thus, indicating that ethanol was essential to extract out polar compounds. Cordycepin has been noted to exert anti-proliferative activity on cancerous cell lines like human colorectal adenocarcinoma, HT-29 and human colorectal carcinoma, HCT 116 (Lee et al., 2013; Jeong and Choi, 2014). Cordycepin has also been seen to elicit apoptosis in human leukemia cells *via* ROS-mediated caspase pathway. In line of this, presence of cordycepin in CSF1 and CSF2 indicated their potential bioactive properties.

Nucleobases identified in CSF1 and CSF2 extracts were adenine and cytosine, as seen in Figure 3. The markedly better content of these two nucleobases typically coincided with the higher yield and longer extraction time for procuring CSF1, in comparison to CSF2. Adenine plays key role in cellular respiration in higher organisms and cytosine is essential for energy transport (Hasan et al., 2019). Hence, availability of these nucleobases suggested their prospective bioeffects, especially from a hypoxia-protective standpoint.

Flavonoids encompass a class of bioactive compounds occurring in medicinal plants and mushrooms that have established benefits towards improvement of human health (Nijveldt et al., 2001). Specifically, quercetin, gallic acid, ascorbic acid and rutin were selected as the standard flavonoids for identification in *C. sinensis* supercritical extracts due to their reported anti-carcinogenic, anti-mutagenic and anti-oxidative modes of actions. In addition, supplementation of flavonoids in diet of human volunteers has been cited to induce free radical scavenging, blood viscosity lowering and enhancement of function of vital organs (Hoensch and Kirch, 2005). Similar to cordycepin and nucleobases, even flavonoids namely, quercetin, gallic acid and ascorbic acid were quantitated to be more in CSF1 than CSF2 (Figure 4). These being polar compounds were not extracted efficiently in CSF3, which contained only quercetin.

HPTLC analysis clearly revealed that CSF1 had an overall higher content of all the aforementioned bioactive compounds than CSF2. This was possibly due to the fact that at a constant temperature, a rise in pressure increased the density of solvent, thus, solubilizing more quantities of flavonoids and nucleobases (Miao et al., 2010). A further increase in extraction pressure resulted in decreased vapour pressure which restricted solubility, hence the marginally lower quantities of compounds in CSF2. Nonetheless, presence of bioactive metabolites was confirmed in CSF1 and CSF2 which prompted investigation of their multifarious bioactivities as elaborated later.

VOCs have been extensively studied for diverse bioeffects. The types of VOCs expected in the supercritical CO_2_ extracts in this study were fatty acids, fatty acid methyl esters and trimethylsilyl esters. Similar compounds have been reported in many medicinal plants and fungi (Dey and Chaudhuri, 2016; Adeoye-Isijola et al., 2018). As instance, hexadecanoic acid methyl ester, 9,12-octadecadienoic acid and 11-octadecenoic acid, methyl ester are known to impart anti-inflammatory, hypocholesterolemic and antimicrobial effects (Kalpana et al., 2012). Methyl stearate influences intestinal lipid metabolism regulation and also leads to antinociceptive, antioxidant, antifungal effects and so forth (Adnan et al., 2019). GC-MS analysis of supercritical extracts from *C. sinensis* have unveiled the presence of 9-octadecenoic acid, 9,12-octadecadienoic acid and hexadecanoic acid as the chief VOCs (Jianya et al., 2006).

In the current study, similar VOCs were perceived in CSF extracts (Tables 1–3 and Figure 5). 9,12-octadecadienoic acid (Z,Z)- was the most abundant VOC in CSF1 which is known to possess substantial antimicrobial, antioxidant, diuretic and hepatoprotective properties (Adeoye-Isijola et al., 2018). Also, it is capable of anti-arthritic, anti-histaminic and hypocholesterolemic activities (Kalaivani et al., 2012). In CSF2, 9,12-octadecadienoic acid (Z,Z)-, trimethylsilylester was major VOC which presumedly has diuretic, hypocholesterolemic and skin protectant properties in addition to anti-androgenic and anti-histaminic abilities (Sudha et al., 2013; Abubakar and Majinda, 2016). Finally, CSF3’s most abundant VOC was seen to be hexadecanoic acid, trimethylsilyl ester which has antioxidant and contraceptive-like effects along with antimicrobial and free scavenging activities (Jain et al., 2012; Patil et al., 2013). These well recognized curative properties of constituent VOCs in all three CSF extracts motivated exploration of varied bioactivities of these extracts.

Metabolomics has emerged as an effective approach, especially while standardizing the pharmacological implications of the vast metabolome of natural extracts (Khan et al., 2017). Application of metabolomics by principal component analysis to the chromatographic profiling obtained from HPTLC and GC-MS, deliberated relatedness between the various supercritical extracts. Peak areas and retardation factor values in HPTLC, and area percentages and retention times from GC-MS acted as input data for plotting the variables factor map. PCA works by decomposition of the eigenvector. Mean centring of data and data normalization are integral in this analysis. The primary axis representing the first principal component (“PC”) or Dim 1 is responsible for describing maximum variance in the dataset. The next PC defines rest of the major variance as Dim 2. In present context, an overall variance of 70.85% in terms of metabolites detected by HPTLC was established. For GC-MS, summative variance with respect to metabolites detected, identified or otherwise, was 68.8%. Similarities in metabolite profiling between CSF1 and CSF2, as discussed in “Results” section, was corroborated by the proximal presence of these two extracts in same quadrant of variables factor map (Figure 6).

Extracts from medicinal mushrooms like *Coprinopsis atramentaria*, *Xerocomus chrysenteron*, *Ganoderma lucidum* and *Cordyceps sinensis* possess remarkable antioxidant abilities which is often accredited to their noteworthy content of flavonoids, phenolics, nucleobases and also VOCs (Pal et al., 2015; Bhardwaj et al., 2017). Supercritical CO_2_ fractions from *C. sinensis* have been proven to possess excellent antioxidant effects (Wang et al., 2005). In line of this, the current study brought out efficient free radical (ABTS and DPPH) scavenging and FRAP of all three CSF samples. As reported earlier, flavonoids and VOCs are powerful antioxidant agents and often play key roles in antiviral and anti-proliferative features of natural extracts (Agati et al., 2012; Abubakar and Majinda, 2016). This validated the distinctive antioxidant efficacy of CSF1 followed by CSF2, which was much higher than that in CSF3 (Figure 7). This was plausibly because of higher amount of component polar compounds like flavonoids and nucleobases in the former two extracts due to use of co-solvents (Oman et al., 2013).

Incidence of increased bacterial resistance to synthetic antibiotics and related drugs has set the foreground to survey antibacterial candidates from natural sources (Alves et al., 2012). Supercritical fluid extracts from medicinal sources like *Gingko biloba* and palm are already known to have effective antibacterial and antifungal properties (Akanda et al., 2012; Oman et al., 2013). Concomitant to this, CSF1, CSF2 and CSF3 exerted bactericidal action against *S. typhi* and *E. coli*.

In addition to this, the prevalence of *E. coli* infection has been deemed to be more severe in uropathogenic infections. A study by Lin et al. (2015) had pointed out the importance of transcriptional regulator HIF-1α in innate defense against uropathogenic *E. coli* urinary tract infection. This was relevant to our study since we have used HEK 293 as cell culture model and have elaborated the modulation brought about by CSF extract on HIF-1α. Thus, the concerted effect of *C. sinensis* supercritical CO_2_ extracts in hindering the growth of *E. coli* while normalizing the adverse effects of hypoxia conditions in kidney cell line could be correlated here. As seen in Table 4, CSF1 and CSF2 displayed much better deterrent action with lower MIC values. The antimicrobial property of the *C. sinensis* supercritical extracts can be attributed to the presence of flavonoids like ascorbic acid and gallic acid (Pal et al., 2015). Besides, the constituent VOCs certainly contributed to the noticeable antibacterial activity. In the endeavour to deduce probable modes of antibacterial action, ROS generation within the bacterial cells was observed as a primary mechanism. Amount of ROS generated and hence the degree of bactericidal action, incremented in a dose dependent manner for all the extracts (Figure 8). Recent literature has evidenced the effect of herbal extracts on cellular membrane damage by generating ROS formation in multidrug-resistant *Acinetobacter baumannii* (Zhang et al., 2015; Tang et al., 2016). The dose dependent increase in ROS generation is in accord with previous papers stating that natural compounds act as ROS producers to evoke antimicrobial response (Jiang et al., 2007).

As yet another inhibitory mode, protein leakage in pathogenic bacterial cells was confirmed. The effectiveness of *C. sinensis* supercritical extracts in inducing dose dependent protein leakage is probably indorsed to the richness of these samples in constituent mycoconstituents like flavonoids, nucleobases and bioactive VOCs, as proven from HPTLC and GC-MS experiments. These results are in concurrence to previous reports that flavonoids and VOCs are capable of conferring antimicrobial resistance against pathogenic microorganisms (Abubakar and Majinda, 2016; Singh et al., 2016).

Efflux pumps have been manifested to be major defensive components in Gram negative bacteria by causing active extrusion of antibiotic or related interventions from the periplasm and/or cytoplasm. In this manner, efflux pumps are discerned to render such bacterial pathogens resistant against antibacterial drugs. This type of resistance is often fathomed by the degree of EtBr efflux since viable bacterial cells curb uptake of EtBr *via* active efflux pump inhibitors. As a result, EtBr accumulates in bacterial cells with compromised membranes only, consequently emitting a strong fluorescence. In current study it was seen that CSF2, followed by CSF1 and finally CSF3 revealed efflux pump inhibitory activity against *S. typhi* and *E. coli*, designating their potency against these strains. Relative fluorescence suggesting EtBr accumulation is given in Figure 8F. A study by Lechner et al. (2008) portrays that plant phenolic compounds and flavonoids act as efflux pump inhibitors in *Mycobacterium smegmatis*. Also, secondary metabolites in herbal sources exhibit similar effect (Kumar et al., 2016). Hence, in concurrence to above observations and similar to ROS generation and protein leakage, the bioactive constituents detected in CSF1, CSF2 and CSF3 contributed to the efflux pump inhibition. Afore stated results attest the overall better antibacterial properties of CSF2, trailed closely by CSF1, corroborating the importance of ethanol as a co-solvent while preparing bioactive supercritical extracts.

Various restorative abilities of the medicinal mushroom under consideration have been well elaborated in evidence based medicinal systems. However, there are only few reports that describe the efficacy of *C. sinensis* in countering hypoxia-induced maladies. For instance, treatment with *C. sinensis* aqueous extracts imparted tolerance in adenocarcinoma human alveolar basal epithelial, A549 cell lines against hypoxia by declining ROS generation and lipid peroxidation. Cellular viability was restored up to 52% in the A549 cells. Expression of HIF-1α, NRF-2 and NF-𝜅B were duly tempered, thus, proving the effectiveness of *C. sinensis* in hypoxia tolerance (Singh et al., 2013). Similarly, hydroethanolic extracts of *C. sinensis* endorsed defence against hypoxia in neuronal hippocampal cells HT22, by accentuating cell viability to about 72% and also by enhancing anti-inflammatory cytokines and deterring pro-inflammatory cytokines (Pal et al., 2015). Phenolic fractions from *C. sinensis* conferred appreciable protective action against hypobaric hypoxia milieu in HEK 293 cell lines by recuperating cellular viability up to 79.5%. Additionally, these phenolic fractions catalysed a reduction in levels of superoxide dismutase and oxidized glutathione in experimental male Sprague Dawley rats, within a simulated hypobaric hypoxic setting (Rajput et al., 2020). Owing to above promising results, the *C. sinensis* supercritical CO_2_ extracts were explored for hypoxia protective action in HEK 293 cell line. It was established that CSF2 proved to be a more prospective lead than the other supercritical CO_2_ extracts as well as the aforesaid extracts (aqueous, hydroethanolic and phenolic) to overcome the loss in cellular viability in mammalian cells *in vitro* under low oxygen tension by reviving cellular viability to 82.36% (Figure 9). Bioactive constituents namely, ascorbic acid, rutin as well as adenine atone reduction of cell death *in vitro* under low oxygen stress conditions and therefore, possibly aided the role of CSF2 as therapeutic lead against hypobaric hypoxia (Winter et al., 2016; Patil et al., 2017).

Furthermore, in order to elucidate the mechanism by which CSF2 extract confers protection against hypoxia *in vitro*, its effect in modulating HIF-1α was investigated. HIF-1α, the subunit of HIF-1 transcription factor, is the most important functional component of cellular homeostasis under low oxygen stress that regulates cellular redox status (Nehra et al., 2015). In stark contrast to normoxia conditions, the level of HIF-1α gets overexpressed manifold in oxygen deficient milieu. However, treatment with appropriate therapeutic candidates leads to downregulation of this transcription factor. Congruent results were obtained in current study, where Western blotting analyses explicated that supplementation with optimal dose of CSF2 extensively downregulated the levels of HIF-1α by four-fold thus, establishing its prominent protective role against hypoxic insult in HEK 293 cells (Figure 10).

The major objective of the present study was to establish *C. sinensis* supercritical CO_2_ extracts as prospective therapeutic in a holistic manner. Extracts prepared from medicinal plants and mushrooms consist of a plethora of bioactive compounds. Bio-effects imparted from such extracts are a result of synergistic and/or additive action of various molecules (Zhao et al., 2015; Segneanu et al., 2017; Gupta et al., 2018; Das et al., 2020). This study has confirmed varied activities viz., antioxidant, antibacterial and hypoxia protective action of the supercritical extracts in consideration which largely is because of their richness in terms of different metabolites e.g. flavonoids, nucleobases and VOCs.

## Conclusion

The present research highlighted multifarious pharmacological properties of the well revered medicinal mushroom, *Cordyceps sinensis*. Supercritical fluid extraction using CO_2_ yielded three distinct extracts of superior quality. Elaborate chromatography-based metabolite profiling by HPTLC and GC-MS signposted the rich chemical composition of *C. sinensis*. Application of metabolomics on the chromatographic data by principal component analysis helped in confirming similarities between CSF1 and CSF2. Overall, the results strongly indicate that *C. sinensis* supercritical fluid extracts can be projected as candidates for mycotherapeutics development, especially for recuperation against hypobaric hypoxia. Furthermore, determination of bioprocess efficiency in terms of product quality and yield needs to be addressed to materialize the use of above extracts on an industrial scale.

## Data Availability

The raw data supporting the conclusions of this article will be made available by the authors, without undue reservation.
